# Use of double flaps in pharyngo-laryngo-esophageal reconstructions: a retrospective review

**DOI:** 10.1007/s00405-025-09456-z

**Published:** 2025-05-14

**Authors:** Andrea Sacchetto, Stefano Meneghesso, Marco Mazzola, Luca Sacchetto, Gabriele Molteni, Virginia Dallari

**Affiliations:** 1 Department of Otolaryngology, Ospedale San Bortolo, AULSS 8 Berica, Vicenza, Italy; 2 Unit of Otorhinolaryngology, Head & Neck Department, University of Verona, Piazzale L.A. Scuro 10, 37134 Verona, Italy; 3 Department of Otorhinolaryngology Head and Neck Surgery, IRCCS Azienda Ospedaliero-Universitaria Di Bologna, Policlinico Sant’Orsola-Malpighi, Bologna, Italy; 4 Department of Otolaryngology Head and Neck Surgery, Santa Maria Delle Croci Hospital, AUSL Della Romagna, Ravenna, Italy; 5 Young Confederation of European ORL-HNS, Y-CEORL- HNS, Dublin, Ireland

**Keywords:** Reconstruction, Oncology, Laryngeal cancer, Laryngectomy, Pharyngolaryngectomy, Flap

## Abstract

**Purpose:**

This article aims to review techniques and applications for using double flaps (both free and pedicled, and their combinations) in reconstructing defects from total pharyngolaryngectomies (TPL) or pharyngolaryngo-esophagectomies.

**Methods:**

This systematic review followed PRISMA 2020 guidelines. Three authors screened articles, selecting and extracting data on malignancy characteristics, reconstructive techniques, outcomes and complications.

**Results:**

Eleven articles were reviewed, involving 176 oncologic patients. Most patients (39.8%) had defects in the larynx, hypopharynx and cervical skin, while in 31.8% the double flaps were used to restore the pharynx and protect the visceral anastomosis. In most studies included, preoperative treatments were administered, including radiotherapy (RT), concurrent chemoradiotherapy (CRT), and surgery, either alone or in combination. A wide variety of pedicled and free flaps were described. The most common pedicled flap is the pectoralis flap (81 patients, 46%), while the most used free flap is the jejunum flap (124 patients, 70%).

19 patients (10.8%) manifested partial necrosis or encountered minor complications postoperatively. 5 patients necessitated a surgical revision of the flap.

**Conclusion:**

The literature on surgical reconstructions following TPL or pharyngolaryngo-esophagectomies using double flaps is limited. The use of double flap is indicated in cases of TPL with extensive skin defect but is also recommended in case of salvage TPL without skin defect.

**Supplementary Information:**

The online version contains supplementary material available at 10.1007/s00405-025-09456-z.

## Introduction

Reconstructing defects resulting from total pharyngolaryngectomy (TPL) poses intricate technical challenges due to the frequent involvement of irradiated tissue (salvage TPL) and the prevalence of advanced disease and comorbidities in patients. Consequently, there is a growing apprehension regarding the occurrence of pharyngeal leaks and pharyngocutaneous fistulae [1]. The primary emphasis on wound healing is essential for enabling the timely initiation of potential adjuvant therapy [2]. This is particularly important as patients undergoing these surgical procedures often present with advanced hypopharyngeal-laryngeal cancers, necessitating adjuvant treatment.

The objectives of reconstruction involve restoring gastrointestinal continuity using well-vascularized tissue, improving speech and swallowing functions, and minimizing complications such as fistulas and strictures [1]. Moreover, since survival rates for head and neck oncology patients continue to improve worldwide, it is increasingly important that our reconstructive efforts not only restore function but also support long-term well-being. Ensuring that patients can achieve psychosocial reintegration after reconstruction and maintain a satisfactory quality of life over the long term is crucial [3].

The extensive body of literature on pharyngeal reconstruction highlights the complexity of challenging cases, the variety of reconstructive techniques and their modifications, and the rapid advancements in hypopharyngeal reconstruction [4].

In the literature, there is frequent analysis on the use of a single flap for the reconstruction of defects following TPL or pharyngolaryngo-esophagectomies. The options range from regional myopedunculated flaps and gastric transposition to free intestinal flaps and fasciocutaneous flaps such as Anterolateral Thigh Flap (ALTF) and Radial Forearm Free Flap (RFFF) [4].

However, there are times when extensive composite defects and previous chemoradiation treatment warrant the use of a double flap (free, pedicled, or a combination of both) to mitigate the risk of post-operative complications, which, in certain instances, can escalate to life-threatening conditions. Nevertheless, this major surgery introduces new challenges, such as prolonged operating times, increased stress on a medically vulnerable patient population, and technical difficulties, such as securing an additional set of vessels in previously irradiated tissue. These challenges have led some clinicians to favor the use of a regional flap to augment and enhance the coverage capability of a single free flap [5].

The purpose of this article is to review techniques for employing double flaps in the reconstruction of defects resulting from TPL or pharyngolaryngo-esophagectomies. Additionally, we aim to analyze the reconstruction purposes for which they are used.

## Methods

### Search strategy

This systematic review followed the guidelines outlined in the Preferred Reporting Items for Systematic Reviews and Meta-analysis (PRISMA) 2020, ensuring a scientific research strategy aimed at minimizing bias. The approach involved a systematic compilation, critical evaluation, and synthesis of all pertinent studies published on the selected topic [6]. The PICO guidelines and PRISMA flow diagram were used in identifying relevant studies which were assessed using the risk of bias.

In March 2024, a computerized MEDLINE search was conducted using the PubMed service of the U.S. National Library of Medicine (www.pubmed.org) and the Scopus database (www.scopus.com). The search strings used were as follows: ("salvage laryngectomy") OR (laryngectomy) AND (flap); ("salvage laryngectomy") OR (pharyngolaryngectomy) AND (flap); ("salvage laryngectomy") OR (laryngopharyngectomy) AND (flap). The initial search yielded a total of 2130 results.

In June 2024, searches were updated and rerun on MEDLINE, adding the following search strings: ("pharyngoesophageal") AND ("flap"), ("pharyngoesophageal") AND ("reconstruction"), resulting in an additional 409 articles, bringing the total to 2539 articles to be screened.

After removing duplicates, the titles and abstracts obtained were independently screened by three of the authors (S.M, M.M., and A.S.). The same authors then evaluated the full texts of all the relevant articles identified against the inclusion criteria. Any disagreement among the assessors regarding the suitability of articles for inclusion was resolved through extensive discussion among the assessors. If consensus couldn't be reached, the matter was referred to a senior author (G.M.).

**Fig. 1:**
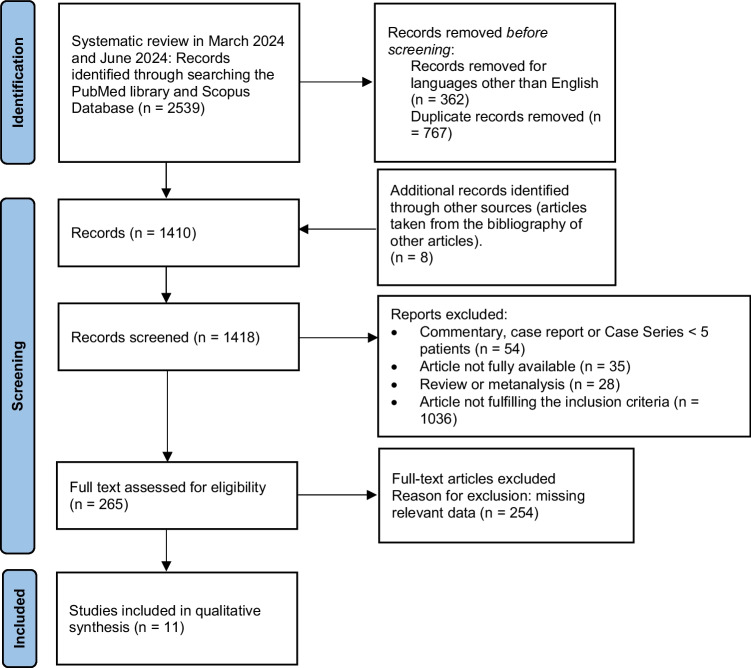
Prisma flow diagram of the review. From: Page MJ, McKenzie JE, Bossuyt PM, Boutron I, Hoffmann TC, Mulrow CD, et al. The PRISMA 2020 statement: an updated guideline for reporting systematic reviews. BMJ 2021;372:n71. doi: 10.1136/bmj.n71

After conducting a manual search of the references from the pooled full texts, we identified the final number of articles included in the present review (Fig. 1). A total of 11 articles were examined.

Subsequently, the main information was extracted and summarized in a database.

## Inclusion criteria

Studies published in the English language, cross-sectional case–control, cohort studies and case series with an adequate number of patients (≥ 5) were included.

Particularly, studies were included if they met the following criteria:

1. Patients underwent total laryngectomy combined with partial or total pharyngectomy with or without cervical esophagectomy;
2. Surgical treatments were performed to treat neoplasms of the laryngeal-pharyngeal-esophageal region;
3. Reconstruction was performed using double flaps, either free or pedicled;
4. Patients over 18 years old;
5. Detailed surgical procedure data available.

## Exclusion criteria

Non-English language studies were excluded. Studies containing aggregated data or duplicated data from previously published work were excluded, as were review articles, case reports with an inadequate number of patients (< 5), editorials, and letters.

All studies where patients undergoing pharyngolaryngectomies or pharyngolaryngo-esophagectomies were reconstructed with a single flap (including bipedicled or bilobed) were excluded. Patients surgically treated for non-oncological reasons were excluded.

Additionally, resections including total glossectomies and extensive esophagectomies, as well as their respective reconstructions, were also excluded.

## Data analysis

Due to the variability in data presentation, a statistical meta-analysis of these results was not feasible. Therefore, the findings were presented descriptively.

## Quality assessment

Two authors (VD, AS) independently evaluated the quality of the included studies using the Newcastle–Ottawa Scale [7].

## Ethical statement

This research was conducted in full accordance with the World Medical Association Declaration of Helsinki (2002). Due to the nature of this study, the Institutional Review Board of the University Hospital of Verona, Italy (Ethics Committee for clinical trials of the Provinces of Verona and Rovigo) does not perform a formal ethical assessment. There was no funding source for this study.

## Results

A total of 11 studies were included in this systematic review, as shown by the PRISMA flow diagram (Fig. 1). All the selected studies had a retrospective analysis design.

An overview of the primary findings of the selected articles can be found in Table 1.

**Table 1:** Overview of the primary findings presented. PMMCF, pectoralis major myocutaneous flap; PMMF, pectoralis major muscle flap; RFFF, radial forearm free flap; ALTF, anterolateral thigh flap; CRT, chemoradiation therapy; RT, radiotherapy

Authors and publication date	Sample size	Tumor site	Previous treatment	Defect type	Flap type	Main findings	Postoperative complications
Chu, 2002 [8]	12	Hypopharynx	None	Circumferential defects of the pharyngoesophageal segment	Laryngotracheal flap + a patch-on PMMCF	The authors found that the laryngotracheal flap can be extended to the fifth and sixth tracheal rings with reliable survival. This is at a level 4 cm below the cricopharyngeal sphincter	Only two minor complications were found, including one hematoma over the donor site of the PMMCF and one local wound abscess
Antohi, 2003 [9]	16 (36 in total)	Larynx and hypopharynx	Not available	Partial pharyngoesophageal defects with less then 50% of their circumference combined with skin and soft-tissue defects (26 patients) Circumferential pharyngoesophageal defects combined with skin and soft-tissue defects (10 patients)	RFFF + deltopectoral flap; Scapular flap + deltopectoral flap; RFFF + PMMCF; Jejunum + PMMCF	The best results in this series were achieved using a combined latissimus dorsi musculocutaneous and scapular cutaneous flap for reconstruction	3 patients required re-explorations because of arterial thrombosis. 1 patient died because of postoperative pneumonia. Other major complications included respiratory tract complications (3) and cardiovascular complications (2). Minor complications included local wound-healing problems which occurred in 11 patients, and fistula in 9 patients (6 of which healed spontaneously)
McCarthy, 2004 [10]	5 (7 in total)	Recurrent pharyngoesophageal carcinoma with stomal recurrence	All 5 patients underwent previous surgery, 4 of them were irradiated	Circumferential defects of the pharyngoesophageal segment and cutaneous resurfacing and tracheostomal reconstruction	Jejunum free flap + deltopectoral flap	The deltopectoral flap provides a large surface area of well vascularized tissue that provides reliable coverage of the newly reconstructed cervical esophagus and exposed major vessels following exenteration of the central compartment. This regional flap is easily contoured to fit peristomal defects and defects of surrounding cervical skin	One patient developed a recurrent pharyngocutaneous fistula that persisted despite conservative treatment. On postoperative day 51, this patient underwent re-resection of the fistula and closure of the resultant defect with a contralateral PMMCF
Watanabe, 2007 [11]	6	Recurrence of hypopharyngoesophageal carcinoma	3 patients underwent previous CRT, 3 patients only RT	Circumferential defects of the pharyngoesophageal segment and cutaneous resurfacing and tracheostomal reconstruction	Jejunum free flap + deltopectoral flap	Simultaneous cervical skin reconstruction with a deltopectoral flap, after sacrificing the irradiated anterior cervical skin, was performed with epidermization of the donor site of the flap with a graft from the femoral area	In 2 cases, stenosis of the tracheostoma was observed because of the coverage of the stoma by the sagging deltopectoral flap; this was treated by tube insertion or pulling up of the sagging skin with a tape after hospitalization
Dubsky, 2007 [12]	8	Recurrent hypopharyngeal carcinoma	1 patient underwent previous surgery and CRT, 1 patient underwent surgery and RT, 1 patient received only RT, and 5 patients underwent previous concurrent CRT	Circumferential defects of the laryngopharyngeal segment	Jejunum free flap + PMMF	To cover the jejunum free flap, intestinal anastomoses, and exposed great vessels of the neck with nonirradiated well- vascularized tissue, a PMMF was harvested. In all cases, the bulk of PMMF made primary cervical closure impossible. A meshed skin graft taken from the thigh was used to cover the exposed muscle bulk	Only 1 patient underwent successful dilation in an outpatient setting and did not require repetitive interventions
Andrades, 2008 [13]	11 (30 in total)	Primary or recurrent hypopharyngeal carcinoma	Not available	Complex neck resection including hypopharynx, larynx, cervical esophagus, and skin (composite neck)	Jejunum free flap + RFFF; Jejunum free flap + rectus abdominis flap	The facial, internal mammary and external carotid arteries, and a stump of the internal jugular vein were the most used recipient vessels for the first or inner flap and for the second or external flap. In 7 cases due to extensive hypopharynx and cervical esophagus resection in vessel depleted necks, bilateral internal mammary pedicles were used successfully	Two cases required local flaps for fistula closure, one patient required dilation to treat pharyngoesophageal stenosis. In two cases, an anastomosis re-exploration was needed: one RFFF artery thrombosis and one jejunum vein
Moradi, 2010 [14]	14 (43 in total)	Recurrence of hypopharyngeal carcinoma	All these patients underwent neoadjuvant RT	Circumferential defects of the laryngopharyngeal segment	Jejunum free flap + PMMF	All patients who had been exposed to previous RT received a prophylactic PMMF to cover both bowel anastomoses unless both anastomoses were performed using the stapler All of their leaks occurred with hand-sutured anastomosis and not with the gastrointestinal stapler	Of these 14 patients, none had a pharyngocutaneous fistula. No prophylactic PMMF were performed for the non-RT group
Miyamoto, 2012 [15]	11	Recurrence of laryngeal or hypopharyngeal carcinoma	Primary treatment consisted of concomitant CRT in 7 patients and RT alone in 4	Circumferential defects of the laryngopharyngeal segment	Jejunum free flap + PMMF	Meshed or sheet split thickness skin graft obtained from thoracic incision edge was performed on the reversed PMMF to cover the neck skin defect	Barium swallowing test revealed ‘‘radiological leak’’ in one patient; however, it did not developed pharyngocutaneous fistula and closed spontaneously. No pharyngocutaneous fistula developed in this series
Zhang, 2014 [16]	23	Advanced carcinoma of the hypopharynx, 8 patients had synchronous second primary tumor in the thoracic esophagus	9 patients received preoperative treatments, including induction CT, RT, laryngectomy, and neck dissection	Circumferential defects of the pharyngoesophageal segment	Gastric pull-up + PMMCF	Due to the massive defect after radical resection, a PMMCF was raised as the last step either to restore the intestinal continuity in combination with the gastric flap or to cover the pharyngogastric anastomosis and the exposed great vessels of the neck	2 patients developed anastomotic leakage, that was resolved by surgical intervention using the contralateral PMMCF. Other 11 patients experienced complications that included wound infection and anastomotic stenosis; and none of these complications required surgical intervention
Kondo, 2018 [17]	65 (90 in total)	Larynx and hypopharynx	Patients who had received preoperative RT were excluded	Circumferential defects of the pharyngoesophageal segment and cutaneous resurfacing and tracheostomal reconstruction	Jejunum free flap + bilateral modified deltopectoral flap	The authors use a surgical technique that includes use of a modified deltopectoral flap and entails lifting a pectoral flap from the neck and advancing it towards the neck to prevent tension on the skin and a subcutaneous dead space covering the anastomosis	The patient who developed an anastomotic leak had a minor leak at the site of the pharyngeal–jejunal anastomosis
Zarins, 2019 [2]	5 (26 in total)	Laryngeal or hypopharyngeal carcinoma	Three patients received CRT with curative intent. Two patients underwent surgery as a primary treatment	Circumferential defects of the laryngopharyngeal segment and cutaneous resurfacing	ALTF + pedicled latissimus dorsi flap; ALTF + PMMCF; Jejunum + PMMCF	A pedicled flap was utilized for neck skin resurfacing only for five patients (19%) in conjunction with free flaps	One pharyngocutaneous fistula needed revision surgery. Oesophageal stricture occurred in one patient. None of the free flaps was lost

The total population included 176 individuals, with a mean age of (61.6 ± 4.9 years).

The anatomical defects varied among the patients, with the majority (39.8%) having defects in the larynx, hypopharynx, and cervical skin, followed by 21.6% of the patients who underwent surgical ablation involving the larynx, hypopharynx, cervical esophagus, and cervical skin.

In the remaining cases, cervical skin was preserved, with 19.9% of surgical defects involving the larynx, hypopharynx and cervical esophagus, and 18.7% the larynx and hypopharynx alone.

In most studies included, preoperative treatments were administered, including radiotherapy (RT), concurrent chemoradiotherapy (CRT), and surgery, either alone or in combination. In 54.5% of cases, the preoperative treatment was not specified.

In the studies analyzed, a wide variety of flaps were used, both free and pedunculated, such as: laryngotracheal flap, RFFF, deltopectoral flap, pectoralis major myocutaneous flap (PMMCF), pectoralis major muscle flap (PMMF), scapular flap, latissimus dorsi flap, deltopectoral flap, jejunum free flap, gastric pull-up, ALTF, and rectus abdominis flap, variously combined with each other according to surgical needs. A detailed description of the flap combination used can be found in Table 2.

**Table 2:** Summary of the main results described. PMMCF, pectoralis major myocutaneous flap; PMMF, pectoralis major muscle flap; RFFF, radial forearm free flap; ALTF, anterolateral thigh flap; CT, chemotherapy; CRT, chemoradiotherapy; RT, radiotherapy

Characteristics	Total
Number of studies	11
Study population	176
Age (mean ± SD)	61.6 ± 4.9
Anatomical defect, n (%)	
Larynx + hypopharynx	33 (18.7)
Larynx + hypopharynx + cervical skin	70 (39.8)
Larynx + hypopharynx + cervical esophagus	35 (19.9)
Larynx + hypopharynx + cervical esophagus + cervical skin	38 (21.6)
Preoperative treatment, n (%)	
RT	32 (18.2)
Concurrent CRT	18 (10.2)
Surgery	3 (1.7)
Surgery + RT	5 (2.8)
Surgery + CRT	10 (5.7)
None	12 (6.9)
Not specified	96 (54.5)
Flap type, *n* (%)	
Laryngotracheal flap + PMMCF	12 (6.8)
RFFF + deltopectoral flap	6 (3.4)
Scapular flap + deltopectoral flap	1 (0.6)
RFFF + PMMCF	6 (3.4)
Jejunum free flap + PMMCF	4 (2.3)
Jejunum free flap + PMMF	33 (18.7)
Jejunum free flap + deltopectoral flap	11 (6.2)
Gastric pull-up + PMMCF	23 (13.1)
Jejunum free flap + bilateral modified deltopectoral flap	65 (36.9)
ALTF + pedicled latissimus dorsi flap	1 (0.6)
ALTF + PMMCF	3 (1.7)
Jejunum free flap + RFFF	10 (5.7)
Jejunum free flap + rectus abdominis flap	1 (0.6)
Aim of reconstruction, n (%)	
Restoration of the cervical skin and alimentary tube	38 (21.6)
Restoration of the alimentary tube	12 (6.8)
Restoration of the alimentary tube and coverage anastomoses and exposed great vessels of the neck	56 (31.8)
Restoration of the alimentary tube, coverage anastomoses and exposed great vessels of the neck and restoration of the cervical skin	70 (39.8)
Flap outcomes, n (%)	
Partial necrosis or minor complications	19 (10.8)
Need for revision surgery	5 (2.8)
Reconstruction outcomes, n (%)	
Pharyngocutaneus fistula	16 (9.1)
Pharyngo-pharyngoesophageal strictures	7 (4.0)
Need for revision surgery	10 (5.7)
Feeding, n (%)	
Oral diet	74 (42)
Enteral nutrition	12 (6.8)
Not available	90 (51.2)
Donor site complications, n (%)	
Minor complications	6 (3.4)
Need for revision surgery	0
Postoperative treatment, n (%)	
CT	10 (5.7)
RT	27 (15.3)

In every instance, the primary objective of the reconstruction was the restoration of the alimentary tube. In a subset of these cases (21.6%) restoration of cervical skin was also necessary. A significant proportion of patients (70 cases, 39.8% of the total) required a double flap reconstruction to ensure coverage of anastomoses and the exposed major vessels of the neck, in addition to the previously mentioned indications.

Regarding flap outcomes, 19 patients (10.8%) manifested partial necrosis or encountered minor complications postoperatively, such as local wound dehiscence or infection. A smaller cohort of 5 patients necessitated a surgical revision of the flap.

Regarding the reconstruction outcomes, there were 16 cases (9.1%) of pharyngocutaneous fistula formation, 7 occurrences (4.0%) of pharyngo-pharyngoesophageal stricture development, and 10 cases (5.7%) that required subsequent revision surgery. Data regarding the type of nutrition in patients undergoing surgery was reported in only 49% of cases. In 74 patients (41.2%), oral feeding was resumed, while 12 patients were described as dependent on enteral nutrition via gastrostomy. Observations at the donor site revealed complications in 6 patients (3.4%), characterized by minor issues, with none requiring additional surgical intervention.

Postoperative adjuvant treatment administered in the studies analyzed included chemotherapy (CT) in 10 cases and RT in 27. A summary of the main results described can be found in Table 2.

A systematic comparison between functional and surgical outcomes of the different reconstructive options was not possible due to the heterogeneity of both the described approaches and the data reporting in the selected articles. However, several authors described satisfactory recovery of swallowing function, with most patients able to resume oral intake within a few weeks after surgery [8, 12, 14, 15].

The most comprehensive data that can be analyzed regarding surgical outcomes (i.e., complications) and functional outcomes (i.e., resumption of oral feeding) pertain to three specific surgical alternatives: jejunum free flap (JFF) + pectoralis major flap (*n* = 37), laryngotracheal flap (LTF) + PMMCF (*n* = 12), and gastric pull-up + pectoralis major flap (*n* = 23). All three alternatives show excellent surgical outcomes: only 2 out of 23 patients who underwent reconstruction with gastric pull-up + pectoralis major flap developed a pharyngocutaneous fistula (PCF), while none of the patients who underwent reconstruction with LTF or JFF developed this complication. All patients who underwent these three treatments showed excellent results in terms of resuming oral feeding. All patients resumed exclusive oral intake except for one patient who underwent reconstruction with JFF and a pectoralis major flap, for whom nutritional supplementation via gastrostomy was required.

## Discussion

In advanced laryngeal or hypopharyngeal carcinomas, a TPL, potentially extending to the cervical esophagus, may be required for surgical radicality, either as a primary or salvage treatment in cases of failure of organ preservation therapy or disease recurrence [2]. Reconstruction is challenging due to extensive tissue removal, often requiring more than one flap [16]. The main goals are to ensure proper healing with vascularized tissue, restore functionality (e.g., oral intake), and achieve satisfactory aesthetics to maintain quality of life [3]. Salvage surgery, however, carries a higher risk of complications such as wound dehiscence, infections, fistulas, stenosis, and skin necrosis [15].

Consequently, the reconstruction phase plays a crucial role in surgical planning. To ensure sufficient tissue for defect reconstruction and optimize surgical outcomes while minimizing complications, reconstructive techniques involving double flaps are often necessary.

According to our data, the most common situation that necessitates the use of two different flaps is the presence of a cervical skin defect in patients undergoing TPL.

Indeed, the majority of patients (39.8%) had defects involving the larynx, hypopharynx, and cervical skin, while 21.6% underwent surgical ablation affecting the larynx, hypopharynx, cervical esophagus, and cervical skin. Such surgery may be required in cases of peristomal recurrence or persistence, particularly in patients who are already undergoing radiation or chemotherapy treatment [10–12]. In the remaining cases, the cervical skin was preserved: 19.9% had defects involving the larynx, hypopharynx, and cervical esophagus, and 18.7% had defects limited to the larynx and hypopharynx.

As shown in our review, a wide range of both free and pedicled flaps were used, including laryngotracheal flap, RFFF, deltopectoral flap, PMMCF, PMMF, scapular flap, latissimus dorsi flap, deltopectoral flap, jejunum free flap, gastric pull-up, ALTF, and rectus abdominis flap, often combined depending on surgical requirements.

The flap used for reconstructing the digestive tract is typically either a fasciocutaneous flap (such as ALT or RFFF) or a visceral flap (like the jejunum flap or gastric pull-up). Fasciocutaneous flaps are often preferred due to their pliability and thinness. However, there is a notable risk of peripheral tissue damage, particularly if the flap needs to be tubulized. In contrast, visceral flaps are not suitable for reconstructing exposed areas, such as the cervical skin. For this purpose, a myocutaneous flap is often used, with the deltopectoral or pectoral flap being the most common options, as they are capable of covering extensive defects in the cervical region.

Since the selected articles do not describe the size of the cervical skin defect, it was not possible to identify a specific dimensional threshold that defines the absolute necessity for reconstruction with a double flap. In the planning of reconstruction, however, it is necessary to consider the usual dimensions of the pharyngo-laryngeal region: width of the base of the tongue (range 6–9 cm); and height of the vertical hypopharyngeal defect (range 7–11 cm) [18]. These dimensions may be significantly larger in the case of circumferential pharyngeal resection, extension of the resection to the base of the tongue, or the cervical esophagus. Furthermore, in the case of a skin defect, the size of the defect will also be calculated, along with a portion of the flap that is de-epithelialized, with dimensions varying depending on the geometry of the reconstruction. As a result, if the repair of the complex surgical defect is performed with a single flap, it may in some cases need to be larger than 10 cm in height and 25 cm in length.

Other researchers, such as Moradi and Dubsky [12, 14], have employed a second reconstructive flap during TPL to protect the visceral anastomosis and reduce postoperative complications. Their studies showed that using a pectoral flap alongside a jejunum flap for TPL resulted in a significantly lower rate of anastomotic leaks.

In a similar case series, Zhang et al. [16] successfully combined the gastric pull-up with a pectoralis major flap to restore intestinal continuity or to cover the pharyngogastric anastomosis and the exposed great vessels in the neck.

While the systematic use of additional vascularized tissue to support visceral anastomosis and suture is not standard practice, it may prove particularly beneficial for patients undergoing RT or CRT treatments, as also highlighted by Miyamoto and others [15, 19, 20].

The impact on the reconstructive outcome of any prior RT or CRT treatments before surgery is extremely significant [21]. This information was described in detail only in a limited number of cases (45.5%). However, considering the patients for whom this data was available (*n* = 80), it was observed that 46% had undergone pre-surgical RT (with or without surgery), and 35% had undergone CRT (with or without surgery). This underlines the clinical reality in which many reconstructions are performed in previously treated, high-risk fields. Only 15% had not received any treatment before the surgical procedure with the double flap analyzed.

Previously operated fields in the head and neck show distorted tissue planes, scarring, and impaired vascular and lymphatic pathways, leading to hypo-perfusion, high tension, and increased healing complications. Moreover, the presence of microorganisms in the upper digestive tract heightens the risk of infection and fistula formation [22, 23]. CT and RT induce widespread effects, leading to fundamental cellular changes. Key biochemical responses to radiation in tissues include inflammation, hypercoagulability, and fibrosis [22]. In irradiated head and neck vessels, there is an increased presence of inflammatory cells within the intima [24]. Tissue changes, including vessel depletion and loss of anatomical landmarks caused by prior treatments, add complexity to subsequent surgeries and reconstructions, leading to increased operative time, surgical difficulty, and complication rates. In this context, the use of the double flap technique becomes particularly relevant.

In our review, both pedicled and free flaps are utilized and combined in various ways. Pedicled flaps have traditionally been the cornerstone of head and neck reconstruction due to their reliability and technical simplicity. Meanwhile, free tissue transfer has become the gold standard for salvage surgery reconstruction, with its techniques continuing to evolve. Its use has been well-documented, even in challenging cases such as multiple rounds of radiation or depleted recipient vessels [22]. Wallace et al. reported a 97% success rate for microvascular flaps, underscoring their increasing applicability in reconstructive surgery [3].

Reconstruction of the esophago-pharyngo-laryngeal region is particularly challenging in the case of circumferential defects of the pharynx associated with skin defects. In such cases, the restoration of continuity of the digestive tract is achieved through a tubulized flap, which must be combined with an excess of reconstructive tissue or a second flap to repair the cervical skin defect. In the case of only circumferential defects, the most commonly used flaps are the tubed pectoralis muscle myocutaneous flap (t-PMMCF), the tubed anterolateral thigh flap (t-ALTF), the tubed radial forearm free flap (t-RFFF), the jejunum free flap, and the U-shaped pectoralis muscle myocutaneous flap (u-PMMCF) [25]. Even in cases of reconstruction not associated with a skin defect and in the absence of previous treatments, pharyngocutaneous fistula rates are variably reported between 10% (for jejunum free flap) and 42% (for t-PMMCF), highlighting the potential challenges of this reconstruction [25].

To overcome these challenges, the ALTF has been identified by some authors as one of the most viable options for reconstructing the pharyngolaryngeal region, even when accompanied by a concurrent cervical skin defect. For instance, Tan et al. [26] employed this flap (tubed or inlay) in 5 patients for both pharyngeal and skin reconstruction, including cases with cervical defects larger than 10 cm. Surgical revision was required in one of these patients (20% of complication rate). While this reconstructive option shows promise, it would benefit from a larger case series to further establish its reliability in patients who are at high risk of complications.

Although the use of double flaps may initially seem to present more disadvantages compared to a single flap, this perception is not strongly supported in literature. While double flaps involve harvesting tissue from two donor sites instead of one, this approach allows for smaller, more localized harvests, which can also reduce complications related to donor site closure. Additionally, the use of two flaps offers greater adaptability for addressing extensive or complex defects, as it enables more precise reconstruction tailored to the specific tissue requirements. In contrast, a single large flap might necessitate a more complex design to accommodate multiple tissue types [3]. Finally, the use of double flaps does not appear to significantly prolong operating times, provided there is adequate intraoperative coordination and planning [2, 3].

The limited number of articles available in the literature focusing on the use of double flaps in reconstructive surgery of the head and neck region primarily address cases involving resections that encompass multiple tissue types (bone, mucosa, skin, and soft tissue), such as surgical defects of the oral cavity [5, 13]. In contrast, our systematic review focuses exclusively on the use of double flaps in the reconstruction of TPL, whether salvage or not, with potential extension to portions of the esophagus. To date, no similar or specific reviews are available in the literature.

The application of these reconstructive techniques has expanded the feasibility of surgical treatment even in cases of highly extensive neoplasms. The success and complication rates reported in the selected studies indicate that the use of double flaps in this context is a viable therapeutic option for addressing particularly extensive defects, without significant increase in postoperative complications. Indeed, regarding flap outcomes, 19 patients (10.8%) experienced partial necrosis or minor postoperative complications, such as local wound dehiscence or infection. Notably, only a smaller cohort of 5 patients required surgical revision of the flap (2.8%).

A significant limitation of all the studies included is their retrospective design. Both qualitative and quantitative parameters were not systematically collected across all studies, making it impossible to conduct a rigorous meta-analysis.

Functional recovery data, though inconsistently reported, showed promising outcomes in selected series. Chu et al. [8] reported full oral feeding in all 12 patients, while Dubsky et al. [12] noted resumption of near-normal diet within 17 days on average. Similarly, Moradi et al. [14] found that 66% of patients tolerated a regular or pureed diet, and Miyamoto et al. [15]. reported oral diet resumption in 91% of cases within three weeks. These findings underline the potential of double flap strategies to restore not only anatomical continuity but also swallowing function.

**Fig. 2:**
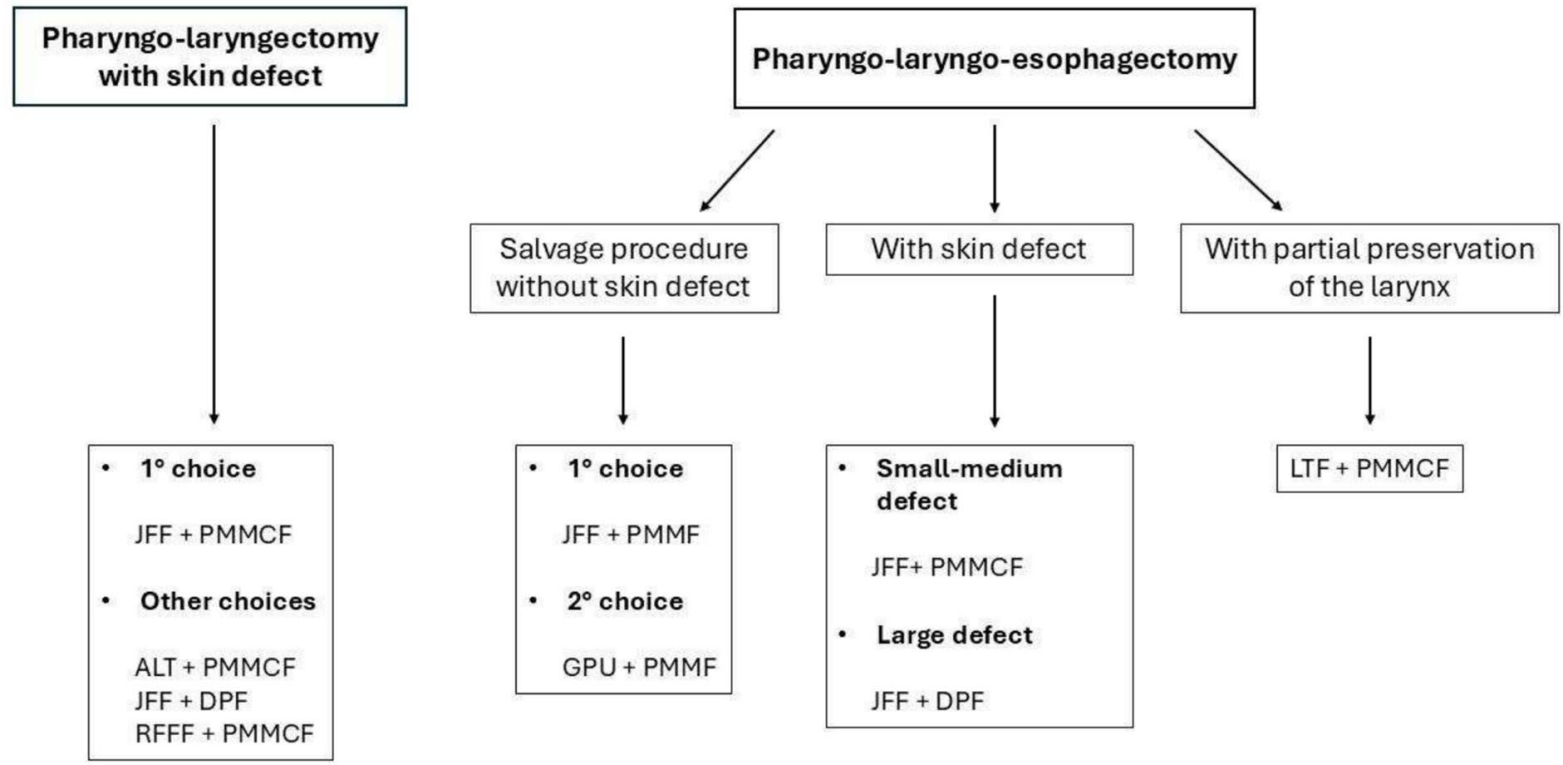
Flowchart of the suggested reconstructive procedure according to the type of ablative surgery following TPL or salvage TPL, with or without cervical esophagectomy. JFF: jejunal free flap; PMMCF: pectoralis major myocutaneous flap; ALT: anterolateral thigh flap; DPF: deltopectoral flap; RFFF: radial forearm free flap; PMMF: pectoralis major myofascial flap; GPU: gastric pull-up; LTF: laryngotracheal flap

As expected in a setting with complex surgical defects and patient comorbidities, significant heterogeneity was observed in reconstructive strategies across the included studies. This variability reflects the absence of a universally accepted standard and highlights the importance of institutional experience and case-specific planning. Nevertheless, the repeated use of certain combinations, such as jejunal free flap with PMMF or PMMCF, suggests emerging patterns of practice. To improve clinical applicability, we developed a decision-making flow-chart (Fig. 2) summarizing the main reconstructive indications emerging from the literature. Although the data remain heterogeneous, the flow-chart integrates common clinical variables such as skin involvement, prior treatment, and reconstructive goals, offering a visual synthesis to support surgical planning.

According to the available literature, there are few articles that focus on and analyze surgical reconstructions following a TPL or salvage TPL using a double flap. As we observed in our review, there are various reasons for opting for a double flap instead of a single one, without a true consensus among different authors. In the authors'opinion, the use of two different reconstructive flaps is strongly recommended in the case of extensive cervical skin resections associated with TPL. However, the use of vascularized tissue is also suggested in patients undergoing salvage TPL, in order to improve the surgical outcome in this category of patients at high risk of failure. Further studies with larger sample sizes and longer follow-up periods are required to confirm and strengthen these findings.

## Conclusion

The use of double flaps in reconstructive surgery for extensive defects following TPL, with or without esophageal extension, represents a promising therapeutic approach. Our systematic review highlights the feasibility and effectiveness of this technique, with favorable success rates and manageable complication profiles reported in the analyzed cases.

This method broadens the scope of surgical options for managing extensive neoplasms and provides reliable solutions for complex reconstructive challenges. In irradiated regions, where prior treatments result in tissue changes, vessel depletion, and loss of anatomical landmarks, the double flap technique is particularly effective. It ensures robust coverage of anastomoses and exposed major neck vessels, reduces the risk of postoperative complications such as fistulas, and restores the alimentary tract and cervical skin when required.

Despite these encouraging outcomes, further high-quality studies are needed to validate these findings, refine surgical protocols, and enhance functional and clinical results for patients undergoing this advanced reconstruction.

## Supplementary Information

Below is the link to the electronic supplementary material.

Supplementary file1 (DOCX 48 KB)

## Data Availability

Data availability statement is not applicable for this review.
