# Supplementary material for: Use of double flaps in pharyngo-laryngo-esophageal reconstructions: a retrospective review
Source: Eur Arch Otorhinolaryngol. 2025 May 14;282(11):5485–96. doi: 10.1007/s00405-025-09456-z (PMC12605531; doi:10.1007/s00405-025-09456-z)
Supplement: Supplementary file 1 — Supplementary file1 (DOCX 48 KB) [file 405_2025_9456_MOESM1_ESM.docx]

Records removed *before screening*:

Records removed for languages other than English (n = 362)

Duplicate records removed (n = 767)

Systematic review in March 2024 and June 2024: Records identified through searching the PubMed library and Scopus Database (n = 2539)

**Identification**

Additional records identified through other sources (articles taken from the bibliography of other articles).
(n = 8)

Records (n = 1410)

Reports excluded:

- Commentary, case report or Case Series < 5 patients (n = 54)
- Article not fully available (n = 35)
- Review or metanalysis (n = 28)
- Article not fulfilling the inclusion criteria (n = 1036)

Records screened (n = 1418)

**Screening**

Full-text articles excluded

Reason for exclusion: missing relevant data (n = 254)

Full text assessed for eligibility

(n = 265)

Studies included in qualitative synthesis (n = 11)

**Included**

*From:*  Page MJ, McKenzie JE, Bossuyt PM, Boutron I, Hoffmann TC, Mulrow CD, et al. The PRISMA 2020 statement: an updated guideline for reporting systematic reviews. BMJ 2021;372:n71. doi: 10.1136/bmj.n71
